# The newly described Araguaian river dolphins, *Inia araguaiaensis* (Cetartiodactyla, Iniidae), produce a diverse repertoire of acoustic signals

**DOI:** 10.7717/peerj.6670

**Published:** 2019-04-19

**Authors:** Gabriel Melo-Santos, Angélica Lúcia Figueiredo Rodrigues, Rodrigo Hipólito Tardin, Israel de Sá Maciel, Miriam Marmontel, Maria Luisa Da Silva, Laura Johanna May-Collado

**Affiliations:** 1 Sea Mammal Research Unit/Scottish Oceans Institute, University of St Andrews, St Andrews, Scotland, United Kingdom; 2 BioMA—Biology and Conservation of Amazonian Aquatic Mammals, Belém, Pará, Brazil; 3 Graduate program in Theory and Research of Behavior, Universidade Federal do Pará, Belém, Pará, Brazil; 4 Research Group on Amazonian Aquatic Mammals, Mamirauá Institute for Sustainable Development, Tefé, Amazonas, Brazil; 5 Laboratory of Ornithology and Bioacoustics, Universidade Federal do Pará, Belém, Pará, Brazil; 6 Department of Ecology, Universidade do Estado do Rio de Janeiro, Rio de Janeiro, Brazil; 7 Laboratory of Bioacoustics and Cetacean Ecology, Universidade Federal Rural do Rio de Janeiro, Rio de Janeiro, Brazil; 8 Graduate Program in Animal Biology, Universidade Federal Rural do Rio de Janeiro, Rio de Janeiro, Rio de Janeiro, Brazil; 9 Department of Biology, University of Vermont, Burlington, VT, United States of America

**Keywords:** Boto, Behavior, Acoustic communication, Mother-calf pairs, Non-linear phenomena

## Abstract

The recent discovery of the Araguaian river dolphin (*Inia araguaiaensis*) highlights how little we know about the diversity and biology of river dolphins. In this study, we described the acoustic repertoire of this newly discovered species in concert with their behaviour. We analysed frequency contours of 727 signals (sampled at 10 ms temporal resolution). These contours were analyzed using an adaptive resonance theory neural network combined with dynamic time-warping (ARTwarp). Using a critical similarity value of 96%, frequency contours were categorized into 237 sound-types. The most common types were emitted when calves were present suggesting a key role in mother-calf communication. Our findings show that the acoustic repertoire of river dolphins is far from simple. Furthermore, the calls described here are similar in acoustic structure to those produced by social delphinids, such as orcas and pilot whales. Uncovering the context in which these signals are produced may help understand the social structure of this species and contribute to our understanding of the evolution of acoustic communication in whales.

## Introduction

Compared to their marine counterparts, sounds produced by river dolphins and their role in communication remain understudied. Mizue, Nishiwaki & Takemura (1971) recorded captive Ganges River dolphins (*Platanista gangetica*) and reported three types of communication sounds: bursts, whistles, twittering and “stratiform” sounds. Other than visualizing spectrograms, no acoustic information was extracted from these sounds. More recently, studies have prioritized the characterization of echolocation (Jensen et al., 2013; Sugimatsu et al., 2017), thus the role of other Ganges dolphin’s sounds remain poorly understood to this date. In the Chinese river dolphin, the baiji (*Lipotes vexillifer*), whistles are thought to be the only communicative sounds produced by the species (Wang, Wenxiang & Zhifan, 1995; Wang, Wang & Akamatsu, 1999; Wang et al., 2006; Xiao & Jing, 1989). The Baiji whistles are lower in frequency (5–6 kHz), less modulated, and produced more infrequently than delphinid whistles (Wang, Wenxiang & Zhifan, 1995; Wang, Wang & Akamatsu, 1999; Wang et al., 2006; Xiao & Jing, 1989). The social sounds of the Franciscana (*Pontoporia blainvillei*) also involved the emission of whistles (1.6–94.6 kHz) as well as burst pulses (Cremer et al., 2017).

Like in all riverine dolphins, the acoustic repertoire of the Amazonian *Inia*, or “boto”, is thought to be restricted to a few types of sounds (Podos, Da Silva & Rossi-Santos, 2002). However, studies of free-ranging and captive botos suggest otherwise. Throughout its distribution several studies have described a variety of sounds including burst-pulsed sounds, jaw-snaps, low-frequency sounds, pulsed sounds, echolocation clicks, and whistles (Amorim et al., 2016; Caldwell, Caldwell & Evans, 1966; Diazgranados & Trujillo, 2002; May-Collado & Wartzok, 2007; Ding, Würsig & Evans, 1995; Ding, Würsig & Leatherwoods, 2001; Kamminga et al., 1993; Podos, Da Silva & Rossi-Santos, 2002; Penner & Murchison, 1970). Ding, Würsig & Leatherwoods (2001) also described the emission of low-frequency whistles (up to 5 kHz) for Peruvian botos. However, this discovery was disputed (Podos, Da Silva & Rossi-Santos, 2002) due to the presence of sympatric tucuxi dolphins (*Sotalia fluviatilis*) known to emit whistles. Later, May-Collado & Wartzok (2007) confirmed that botos do emit whistles, but at much higher frequencies (up to 48 kHz) than previously thought. These high frequency whistles were recorded from botos at the Yasuni and Napo rivers in Ecuador. Today, there is a consensus that, while botos do emit whistles, these sounds are emitted rarely (Diazgranados & Trujillo, 2002; May-Collado & Wartzok, 2007; Ding, Würsig & Leatherwoods, 2001) and likely play a different social role as the one described for delphinids (May-Collado & Wartzok, 2007). Podos, Da Silva & Rossi-Santos (2002) found that the acoustic repertoire of Amazonian botos consisted primarily of pulsed calls with a low emission rate. However, these results were likely limited by the sampling rate of the recorders used by the authors. Amorim et al. (2016) studied the same population using a broadband frequency recording system and described a high emission of a variety of pulsed calls. Botos sounds are relatively more studied than those of other river dolphin species, however much remains to be researched as most of the previous studies were preliminary or localized. While the acoustic behaviour of botos is better documented than those of other river dolphin species, we still do not know their full repertoire nor the role of these sounds in their daily lives.

Botos are evolutionary relics found exclusively in the Amazon, Orinoco, and Tocantins River Basins of South America (Best & Da Silva, 1989; Best & Da Silva, 1993; Hrbek et al., 2014; May-Collado & Agnarsson, 2011; Pilleri & Gihr, 1977; Dos Santos et al., 2012; Dos Santos et al., 2014. Like the franciscana dolphin *(Pontoporia blainvillei)*, the baiji (*Lipotes vexillifer*), and the Ganges and Indus river dolphins *(Platanista* spp.), botos have flexible necks and backbones, a low and large-based dorsal fin, and a slender rostrum (Best & Da Silva, 1989; Best & Da Silva, 1993; Brownell & Herald, 1972; Cassens et al., 2000; Crespo, Harris & González, 1998; Da Silva, 2009; Hamilton et al., 2001; Leatherwoods & Reeves, 1994; Reeves & Martin, 2009; Zhou, 2009). Botos have a preference for habitats with slow currents and high prey concentration such as bays, confluences, small streams, and channels and island margins (Gomez-Salazar et al., 2012; Gomez-Salazar, Trujillo & Whitehead, 2012; Martin & Da Silva, 2004; Martin, Silva & Salmon, 2004; Pavanato et al., 2016). However, residency patterns vary within locations from long-term residency to occasional visitors (Martin & Da Silva, 2004). Although botos are traditionally considered solitary, with long-term social interactions restricted to mothers and their calves, large aggregations have been documented during foraging and mating events (Best & Da Silva, 1989; Best & Da Silva, 1993; Martin, Da Silva & Rothery, 2008). A major constraint in studying river dolphins is that they do not typically perform conspicuous surface displays, making it especially difficult to identify individuals in the field (Best & Da Silva, 1989; Best & Da Silva, 1993; Brownell & Herald, 1972; Cassens et al., 2000; Crespo, Harris & González, 1998; Da Silva, 2009; Hamilton et al., 2001; Leatherwoods & Reeves, 1994; Reeves & Martin, 2009; Zhou, 2009). To overcome these challenges we aimed to study the acoustic repertoire of free-ranging Arauguaian botos (Hrbek et al., 2014) that regularly visit a fish market in Mocajuba in Northern Brazil (Dos Santos et al., 2014; Rodrigues et al., 2019). This semi-controlled setting gave us the unique opportunity to combine acoustic technology with underwater behavioural observations. Our goal was to document the diversity of the acoustic signals produced by these dolphins and the behavioural context in which these signals are produced. As *Inia* acoustic repertoire has been underestimated in the literature (e.g.,  Podos, Da Silva & Rossi-Santos, 2002) we expect to find greater diversity of acoustic signals than what was described so far.

## Material & Methods

### Study area

**Figure 1:**
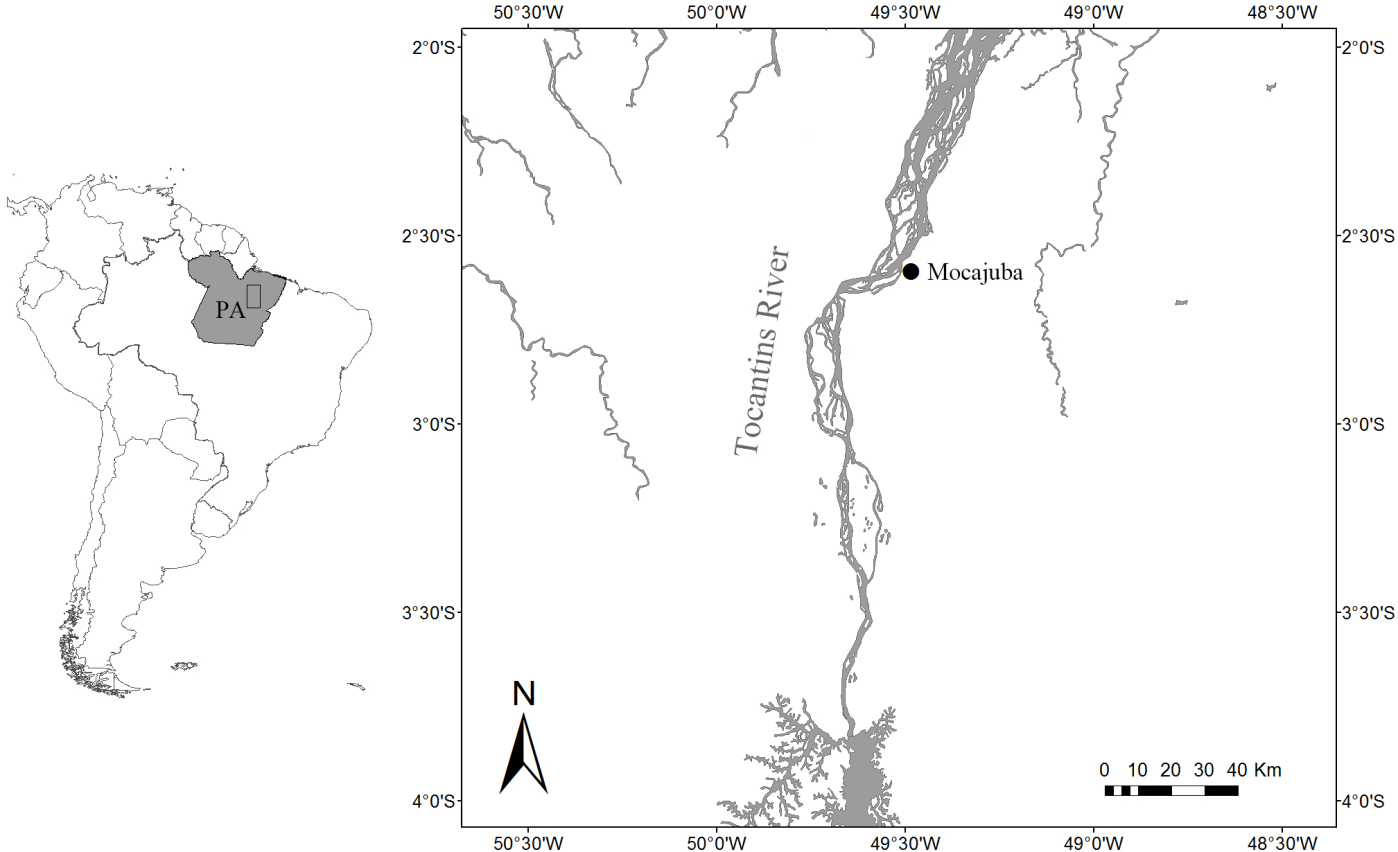
Location of the study area. Location of the Mocajuba fish market at the margins of the Tocantins River.

This study took place along the Tocantins River in the town of Mocajuba in Pará State, Brazil (Fig. 1). The Tocantins River is classified as a clearwater river, it has a small floodplain that crosses through narrow valley. There are large sandbanks in the river’s main channel where herbaceous vegetation may occur, in addition to floating vegetation and submerged aquatic macrophytes where there is light penetration (Junk et al., 2011). At its lower reaches, water cycles are very dynamic with the greatest rainfall from November to April, the highest waters in March, and lowest waters in September (Ribeiro, Petrere & Juras, 1995). There is also a daily cycle of tide pulses (Goulding et al., 2003; Ribeiro, Petrere & Juras, 1995). Mocajuba has a fish market that serves as the main place to acquire fish products for the city and the nearby riverside communities. The wastes of the market and the provision of fish by locals attracts botos to the pier. This set-up together with low turbidity waters during the dry season allows rare proximity to botos, enabling us to identify individuals and observe their behaviour in detail.

### Data collection

Acoustic and behavioural data were collected in visits that ranged from 3 to 15 days during October to December 2013, March 2014, June 2015, July, September and December 2016. The presence of botos at the market depends on the market opening hours, which is the time when the animals are fed (Dos Santos et al., 2014). Therefore, our observations took place in the morning. Behavioural observations were collected in a continuous all-events sampling (Mann, 1999). For each session, we collected the following data: number of individuals present, age class (adult, juvenile, calf), and sex (based on the presence of mammary slits). In addition, animals were identified based on natural marks on the dorsal and ventral parts of the body, given that the botos in the market frequently swim upside down (Dos Santos et al., 2014). Photographs of their bodies were taken with a Nikon 3200 SLR Camera (Nikon Corp., Tokyo, Japan) and a 70 × 300 mm zoom lens (Nikon Corp., Tokyo, Japan). Underwater video was collected with a GoPro Hero 4 (GoPro Inc., San Mateo, CA, USA) held by hand. Notes and drawings of the marks and their locations were also taken if we were unable to take pictures. We held permits to perform this study issued by SISBio (number 52892) from the Brazilian Mistry of Environment.

Sound recordings were taken continuously in synchrony with behavioural observations. We used three recording systems along the study: (1) an Aquarian hydrophone (AFAB Enterprises, Anacortes, Washington, WA, USA) connected to a Tascam DR1 digital recorder (22 kHz sampling rate), (2) a CR1 hydrophone (Cetacean Research Technology, Seattle, WA, USA) connected to a pre-amplifier and a Tascam DR-44WL (96 kHz sampling rate) and (3) a Soundtrap (Ocean Instruments, New Zeland, 576 kHz, sampling rate). Given that *Inia* social sounds are mostly below 48 kHz (May-Collado & Wartzok, 2007) and that the signals found in this study rarely go above 10 kHz (see Results below), we do not believe that the use of different recording systems had an impact in our results. Moreover, the analysis we used here controls for the possible interferences of technical artefacts on sound categorization (see below).

### Data analysis

All recorded signals were inspected using a spectrogram analysis in Raven Pro 1.5 (Cornell Laboratory of Ornithology, New York, NY, USA). Only whistles and pulsed calls with high signal to noise ratio were selected for further analysis. Following this, we extracted the contours from the fundamental frequency of the lowest visible element of the selected sounds using a MATLAB routine called Beluga (https://synergy.st-andrews.ac.uk/soundanalysis/). We then used an adaptive resonance theory neural network combined with dynamic time-warping to group the contours into distinct categories (ARTwarp; (Deecke & Janik, 2006)). This analysis classifies frequency contours according to a critical similarity value or “vigilance” (Deecke & Janik, 2006). In order to account for minimal differences in our classifications we used a high vigilance of 96%. Unlike other methods used to categorize sounds, ARTwarp uses the whole signal while running classifications. In addition, the analysis also allows contours to be shrunk or stretched up to a factor of three, ensuring maximum overlap in the frequency domain when sounds are being compared. This feature may increase the chances of sounds being classified into biologically significant categories (Deecke & Janik, 2006), as patterns of frequency modulation are often more relevant for animal auditory perception than patterns of duration. To reduce the effect of ambient noise and possible technical artefacts from the different recording systems, we re-sampled all frequency contours at 10ms. The analysis was conducted on a MATLAB-based (version 2015b, The Mathworks, Inc.) routine called ARTwarp. Using a rarefaction curve (Magurran, 2004), we evaluated how much of the acoustic repertoire was registered during our sampling period. Using a Whittaker diagram (Magurran, 2004), we assessed the occurrence of the signals recorded as part of these animal’s repertoire. Analyses were conducted in R (version 3.5.1) (R Core Team, 2018).

After this, we made a scatterplot of the minimum versus maximum frequency of the sound types (or “neurons”) resulting from the ARTwarp analysis to estimate the frequency bandwidth of vocalization of Araguaian botos. In addition to the ARTwarp analysis we took information on other characteristics of boto sounds: duration—short (<200ms) versus long (>200ms) signals, and the presence of nonlinear phenomena: (a) subharmonics (signals with additional spectral components in the harmonic stack, generally in multiples of $ \frac{1}{2} $ or 1/3 of the fundamental frequency) and (b) biphonation (signals with the presence of two independent fundamental frequencies) (Tokuda et al., 2002; Wilden et al., 1998).

## Results

**Figure 2:**
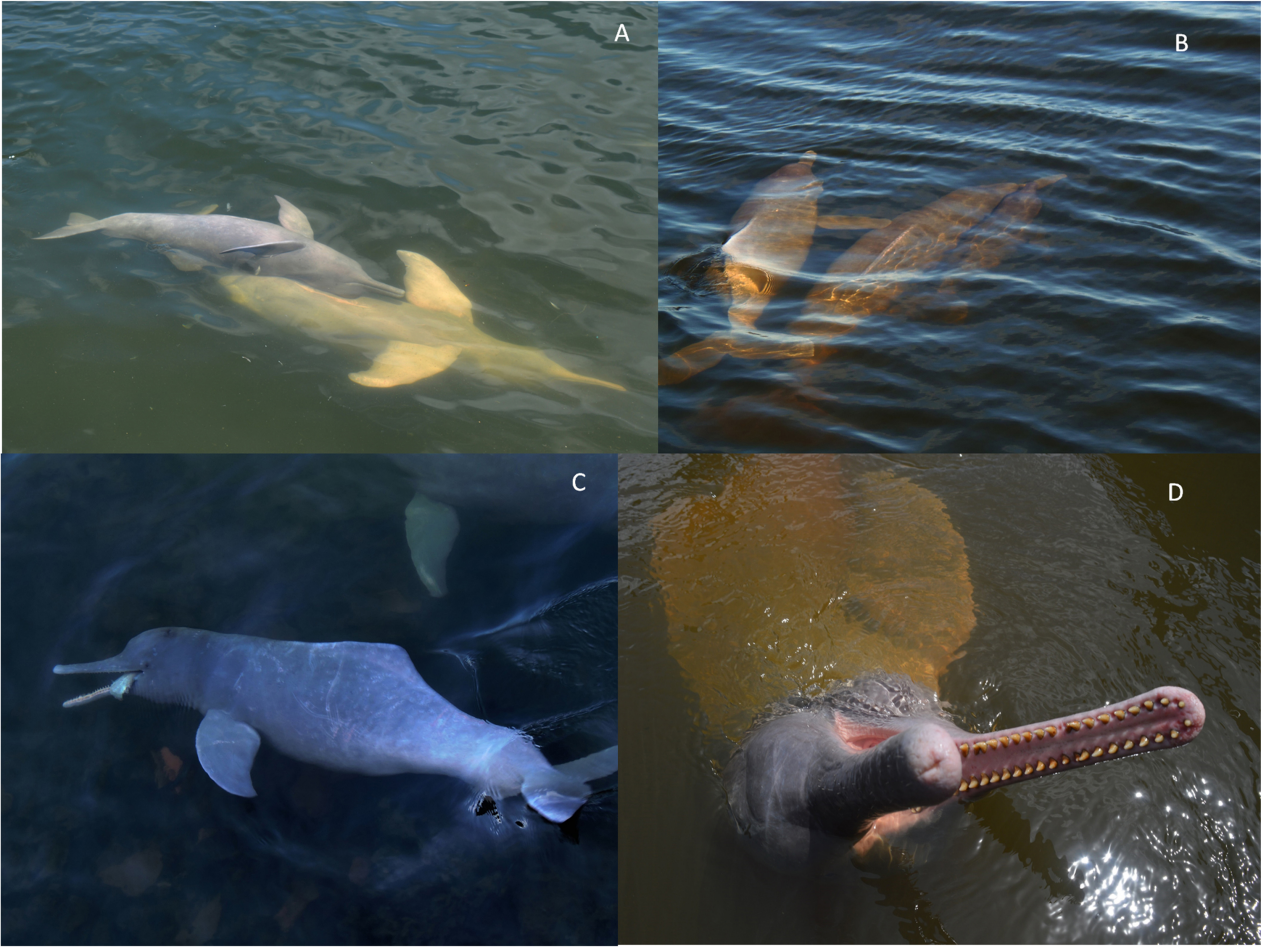
The two behaviours observed during this study. The two behaviours observed during this study were socialization and feeding. (A) and (B): botos engaged in social activity, slow-swimming and physical contact, (C): boto feeding on a fish and (D): waiting to be fed. Photos (A), (B) and (C) by Gabriel Melo-Santos and (D) by Luiza Pereira.

Botos were observed on each of 32 days of data collection effort, resulting in 15.57 h of acoustic recordings. Observed groups ranged between three to 12 individuals. Nine dolphins repeatedly visited the market, allowing us to observe them multiple times. These included five adult females, one adult male, one juvenile female, one female calf and one male calf. The only two behaviours observed during acoustic recordings were socialization and feeding (Fig. 2). Social interactions consisted of animals having physical contact with one another and swimming alongside each other. Occasionally animals would bite the neck of another when waiting to be fed. While we did not test for associations between individuals, the most stable associations appeared to be between mothers and their calves. Feeding behaviour consisted of animals soliciting food with their open-mouthed head out of the water or poking humans with their snout. Furthermore, with the help of underwater cameras we were able to match some of the observations to the vocalizing animals (see below).

**Figure 3:**
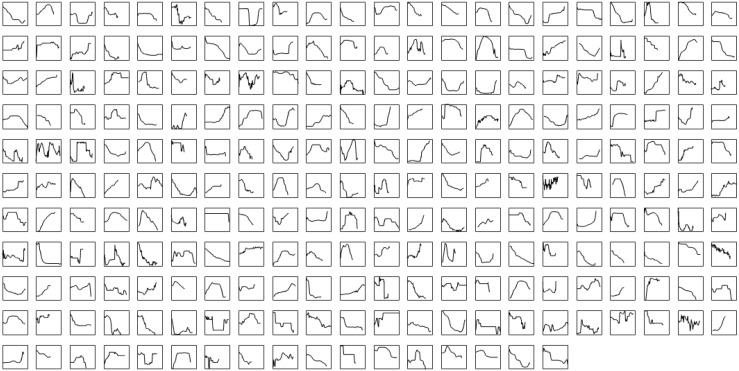
Sound-types produced by Araguaian River dolphins. Frequency contours of the sound-types (neurons) resulting from the ARTwarp analysis for Araguaian botos from the Mocajuba market, Tocantins River.

**Figure 4:**
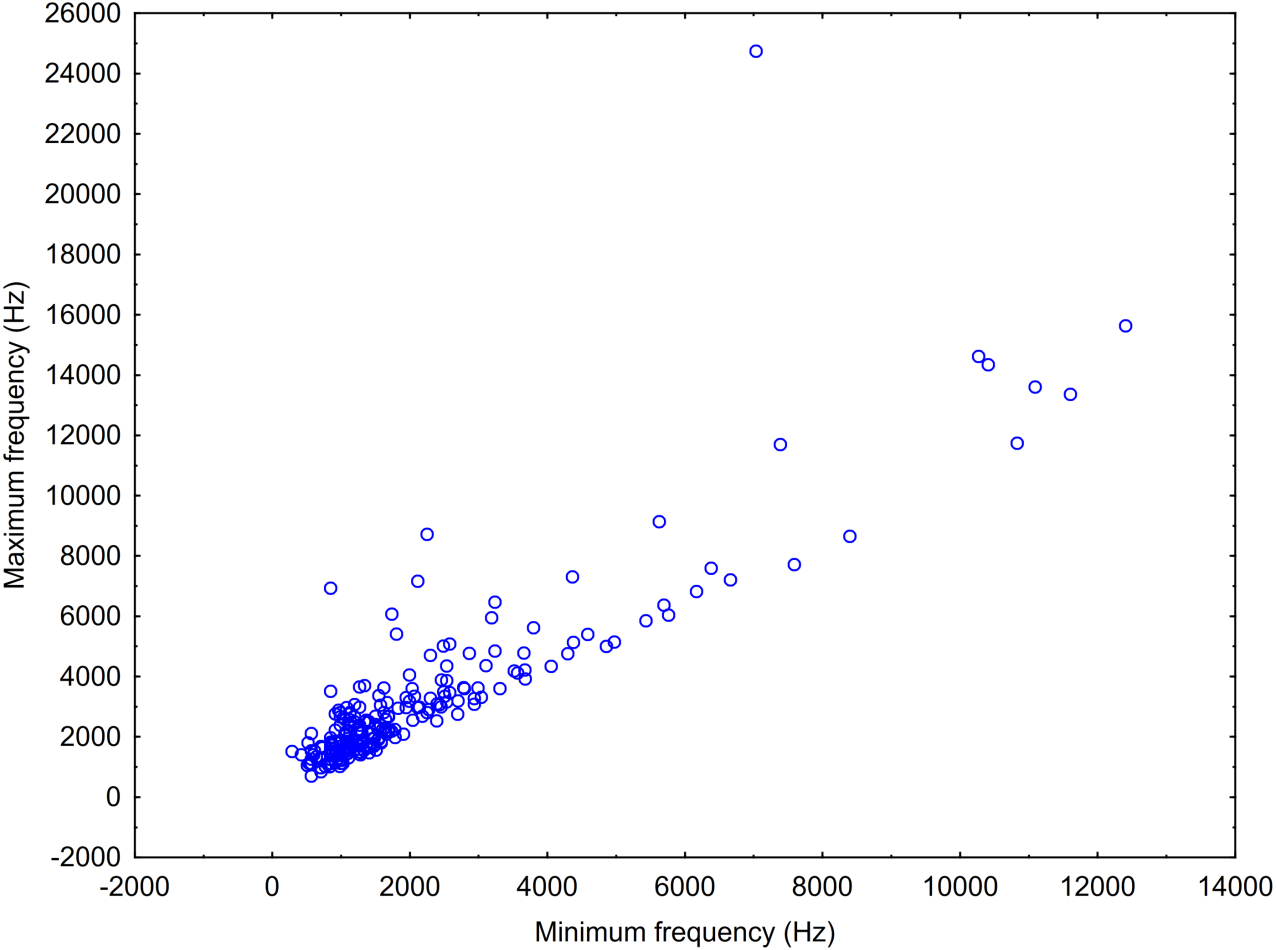
Scatterplot showing the maximum vs minimum frequencies of the neurons resulting from the ARTwarp analysis.

**Figure 5:**
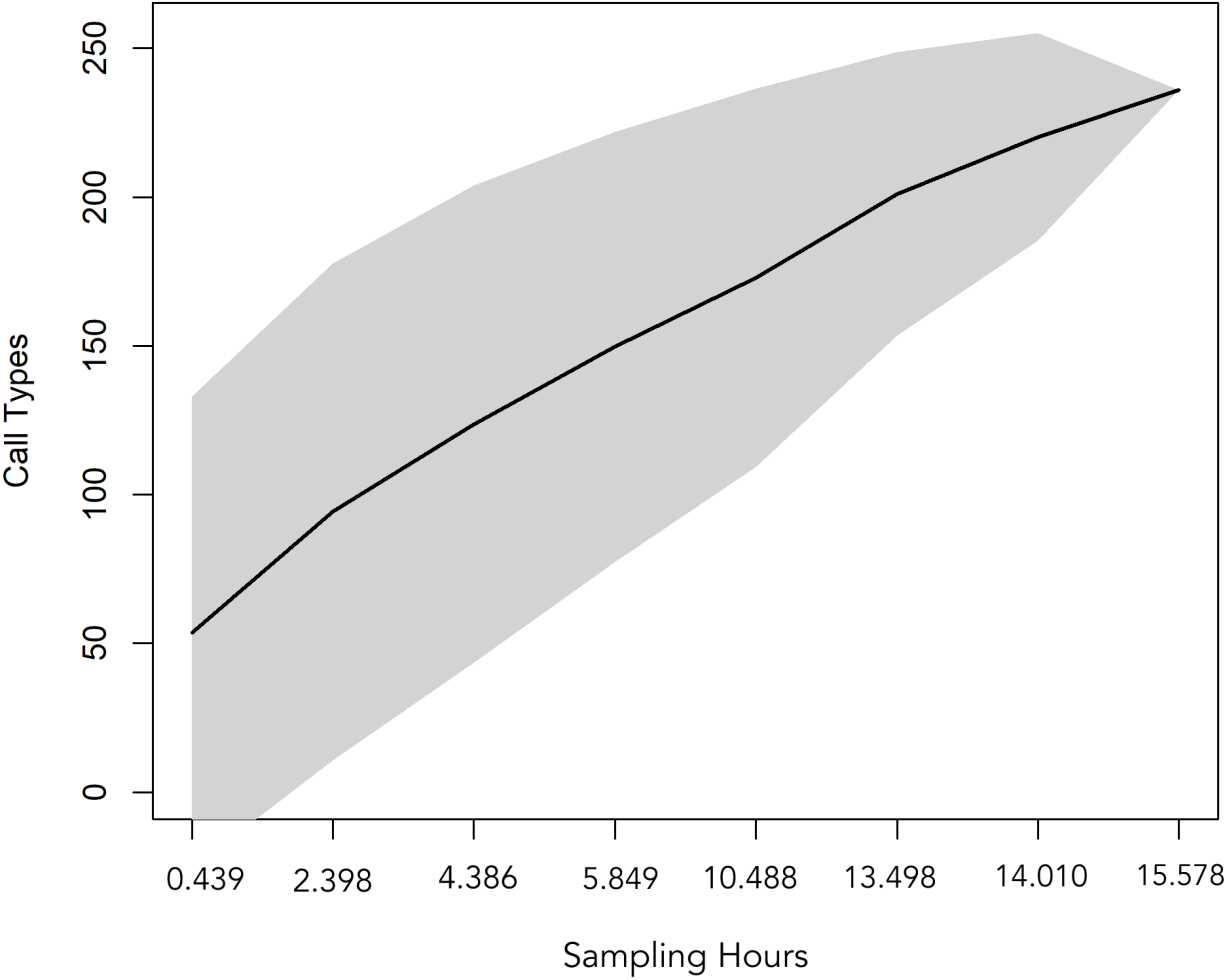
Rarefaction curve showing the cumulative number of sound-types detected with increasing sampling time (hours of recordings analysed). The curve suggests that 15.57 hours of acoustic sampling is not enough to capture most of the acoustic repertoire of the Araguaian river dolphin.

**Figure 6:**
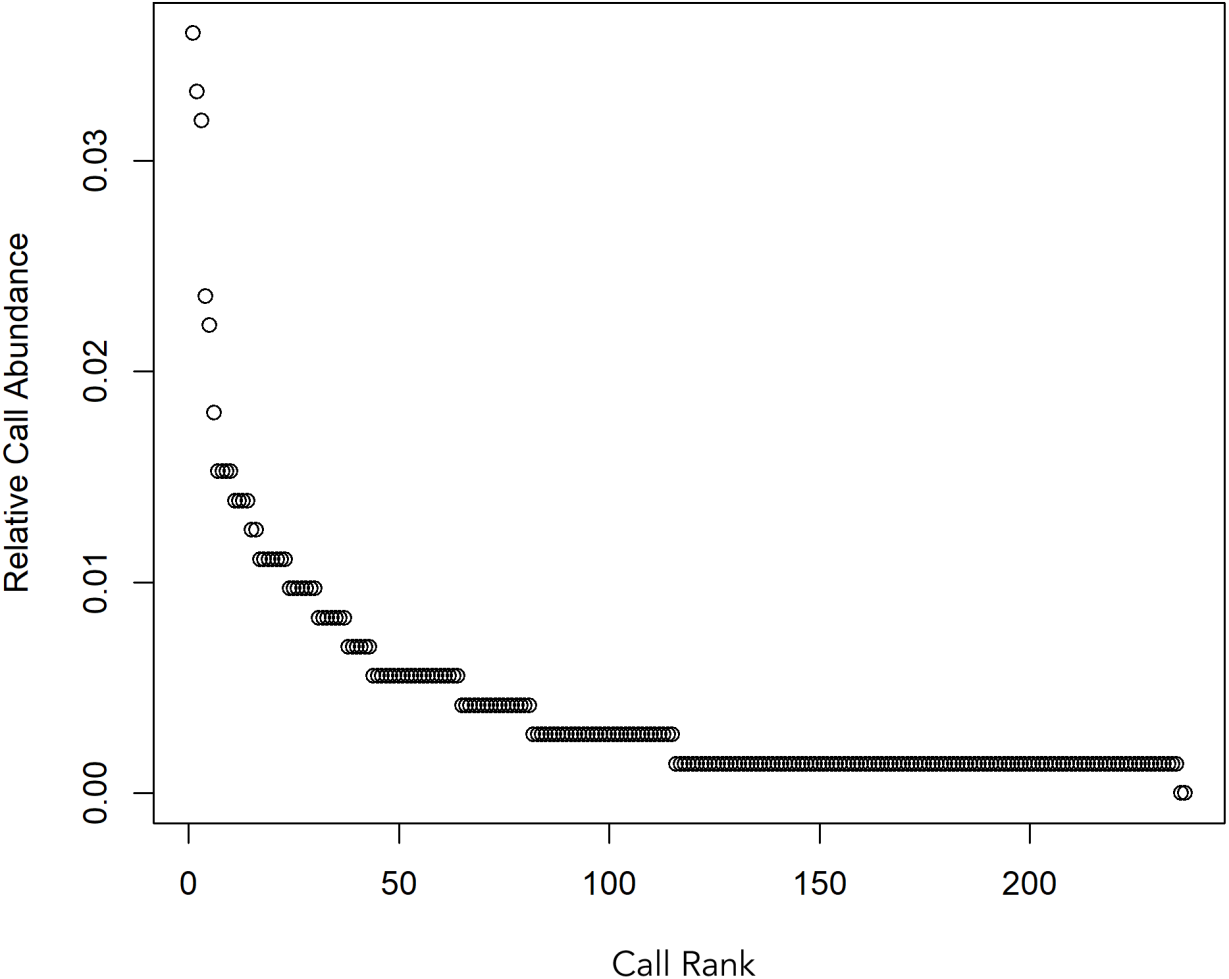
Whittaker diagram displaying the occurrence of sounds emitted by Araguaian botos with most sounds produced rarely.

A total 727 of good quality acoustic signals primarily ranging between 1–10 kHz (Figs. 3 and 4) were used for the ARTwarp analysis, resulting in 237 sound-types. However, the rarefaction curve indicates that our sample was not sufficient to capture most of the acoustic repertoire of these animals (Fig. 5). While there is a great diversity of signals, these botos do seem to produce some signals more abundantly than others (Fig. 6).

**Figure 7:**
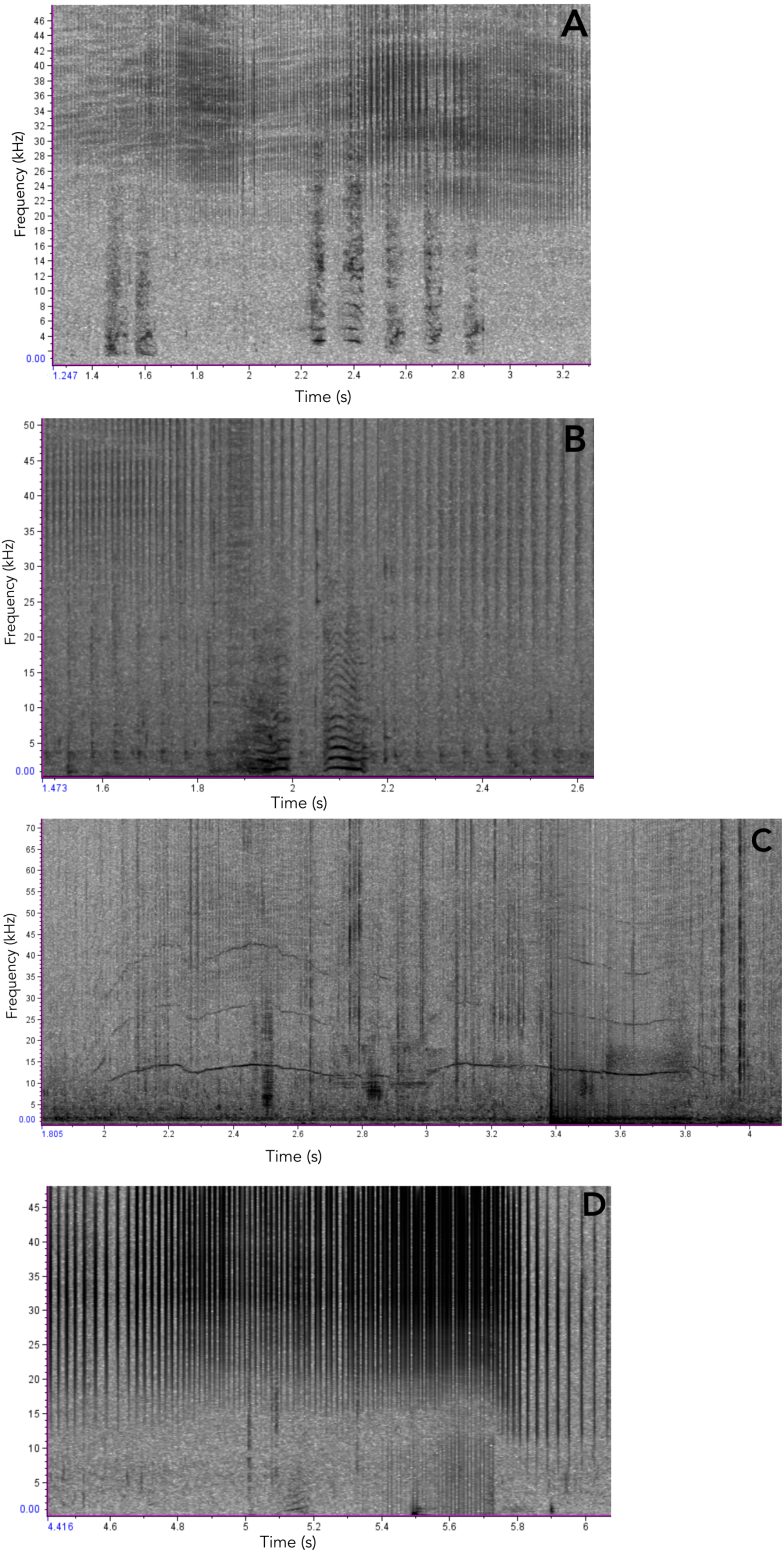
Examples of Araguaian botos acoustic signals recorded during our study. (A) short two-component calls; (B) short calls with subharmonics. (C), narrow-banded frequency modulated whistle and the longest sound registered in this study; (D) Short call with no non-linear phenomena.

**Figure 8:**
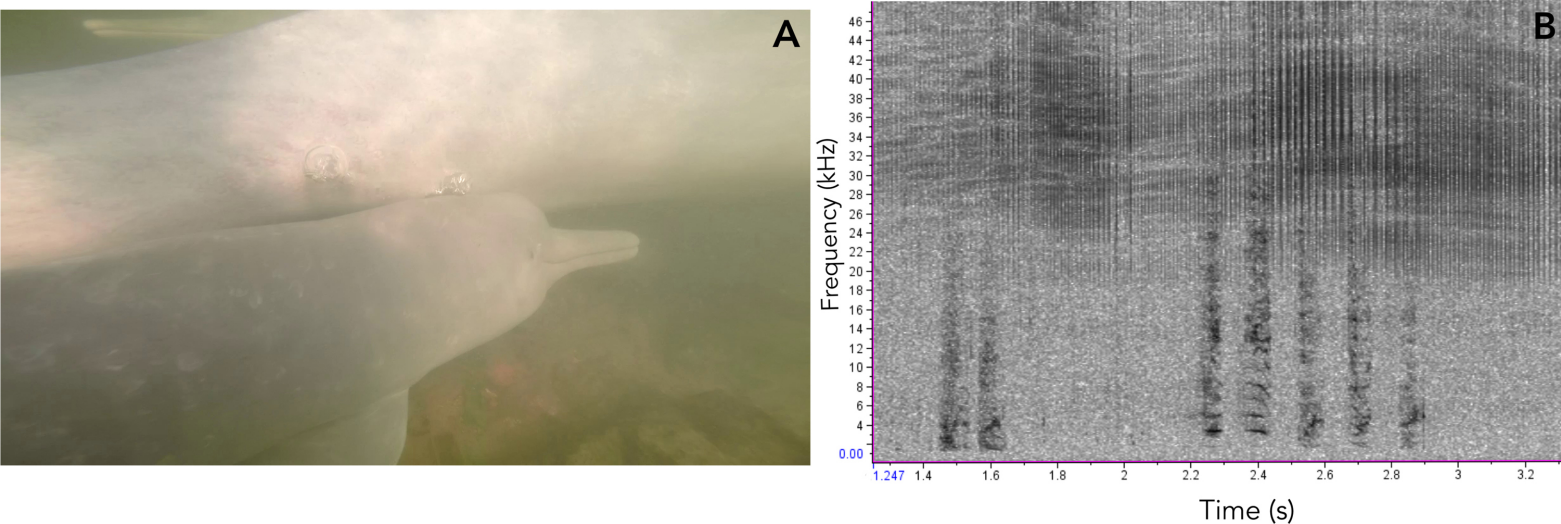
Vocalizations of an Araguaian boto calf. (A) Vocalizing calf as indicated by the bubbles blowing from its blowhole and associated short-two-component call, taken from video footage by Paulo Castro. (B) Spectrogram of calls produced by a calf of Araguaian boto, followed by a bubble stream.

In addition to ARTwarp we also characterized the acoustic signals based on duration and presence of non-linear phenomena. Based on these criteria, botos sounds can be classified into: long-two-component calls, long calls with subharmonics, short calls with biphonation (short-two-component calls), short calls without non-linear phenomena, short-calls with subharmonics, and tonal sounds (Fig. 7, Table 1). The long-calls (*n* = 13; classified into 11 sound-types) and tonal sounds (whistles) (*n* = 21; classified into 18 sound-types) were rarely produced, while the short-two-component calls were the most commonly produced (*n* = 538). Interestingly, 74% (*n* = 538; classified into 184 sound-types) of these were short two-component calls. Underwater video of bubble emission from the blowhole indicate that calves produced short-two- component calls followed by physical contact with their mothers (Fig. 8; Audio S1–S4).

**Table 1:** Classification of sounds recorded from the Araguaian river dolphin at the Mocajuba fish market based on the presence of non-linear phenomena and duration.

Vocalization type	Number of sounds produced
Long two-component calls—pulsed calls longer than 0.200s, with biphonation as the most marking characteristic, may exhibit subharmonics	12
Long calls with subharmonics—calls longer than 0.200s, may exhibit biphonation	1
Short two-component calls—pulsed calls shorter than 0.200s, with biphonation as the most marking characteristic, may exhibit subharmonics	538
Short calls—signals shorter than 0.200s with no non-linear phenomena	53
Short calls with subharmonics—calls shorter than 0.200s, may exhibit biphonation	102
Whistles—tonal narrow-banded signals longer than 0.100s, may exhibit harmonics	21
Total	**727**

## Discussion

Our results show that the Araguaian river dolphin has a more diverse acoustic repertoire than previously documented for the genus *Inia* (Amorim et al., 2016; Caldwell, Caldwell & Evans, 1966; Diazgranados & Trujillo, 2002; May-Collado & Wartzok, 2007; Ding, Würsig & Evans, 1995; Ding, Würsig & Leatherwoods, 2001; Kamminga et al., 1993; Podos, Da Silva & Rossi-Santos, 2002). Early studies described members of the genus *Inia* as silent animals (Podos, Da Silva & Rossi-Santos, 2002) or with a limited acoustic repertoire (Amorim et al., 2016; Caldwell, Caldwell & Evans, 1966; Diazgranados & Trujillo, 2002; May-Collado & Wartzok, 2007; Ding, Würsig & Evans, 1995; Ding, Würsig & Leatherwoods, 2001; Kamminga et al., 1993; Podos, Da Silva & Rossi-Santos, 2002). In contrast, we show that the acoustic repertoire of *Inia* dolphins is diverse and likely as complex as the acoustic repertoire of delphinids.

As described for other *Inia* species, the Araguaina dolphins recorded in our study rarely emit whistles. These findings agree with previous descriptions of whistle emission from Peruvian (Ding, Würsig & Leatherwoods, 2001) and Ecuadorian botos (May-Collado & Wartzok, 2007) and other river dolphins like the Franciscana and the baiji (Cremer et al., 2017; Wang, Wang & Akamatsu, 1999; Wang et al., 2006). In these studies, the function of emitted whistles was unclear. May-Collado & Wartzok (2007) found that botos emitted whistles in a different social context than in delphinids, keeping distance between each other, rather than promoting social interactions as in marine dolphins.

Among pulsed calls, the short-two-component call was the most commonly produced sound. These calls were emitted in what appear to be mother-calf interactions. Our video footage and some underwater follows show bubbles emanating from calves’ blowholes while they emitted these calls as they approached their mothers after a short separation (see Videoes S1 and S2 in DOI: 10.6084/m9.figshare.7992212). Bubble streams are often used as a cue to identify vocalizing animals (Bebus & Herzing, 2015; Fripp, 2005; Jones, 2014) and in this case the bubble stream revealed that the calves were producing the calls and did so in a repetitive fashion. These vocal patterns are similar to what has been described for calves of bottlenose dolphins, which use signature whistle as contact calls, where calves increase whistle emission as they approach their mothers (Smolker, Mann & Smuts, 1993). Given the strength of mother-calf associations in botos (Best & Da Silva, 1989; Best & Da Silva, 1993) and the characteristics of their habitat, a shared signal that enhances mother-calf recognition may be key as they move through murky waters and complex underwater vegetation. The complex structure of botos’ habitat might also have led to evolution towards signals with short duration, longer signals might suffer interference of echoes caused by obstacles (sandbanks, underwater vegetation, riverbed, even the water surface). Notwithstanding, social signals produced by *Inia* sister taxa *Pontoporia* who also evolved in riverine environments are short as well (Cremer et al., 2017). Meanwhile, the frequency bandwidth of Araguaian river dolphins vocalizations are intermediate when compared to delphinids and baleen whales (Au, 2000; Boisseau, 2005; Clark, 1994; Clark, 1995; Lammers, Au & Herzing, 2003; May-Collado & Wartzok, 2009; Tyack, 2000). Araguaian botos’ social sounds are lower in frequency than those of delphinids, though not as low as baleen whale calls. Sounds with lower frequencies should travel greater distances and due to larger wavelength would be able to deviate from possible obstacles in between vocalizing animals (e.g., submerged vegetation, rocks). We hypothesize that because groups o *Inia* are not as cohesive as delphinids, sounds emitted at intermediate frequency range would be more efficient for communication in a complex habitat as rivers. However, further studies are necessary to test this hypothesis.

**Figure 9:**
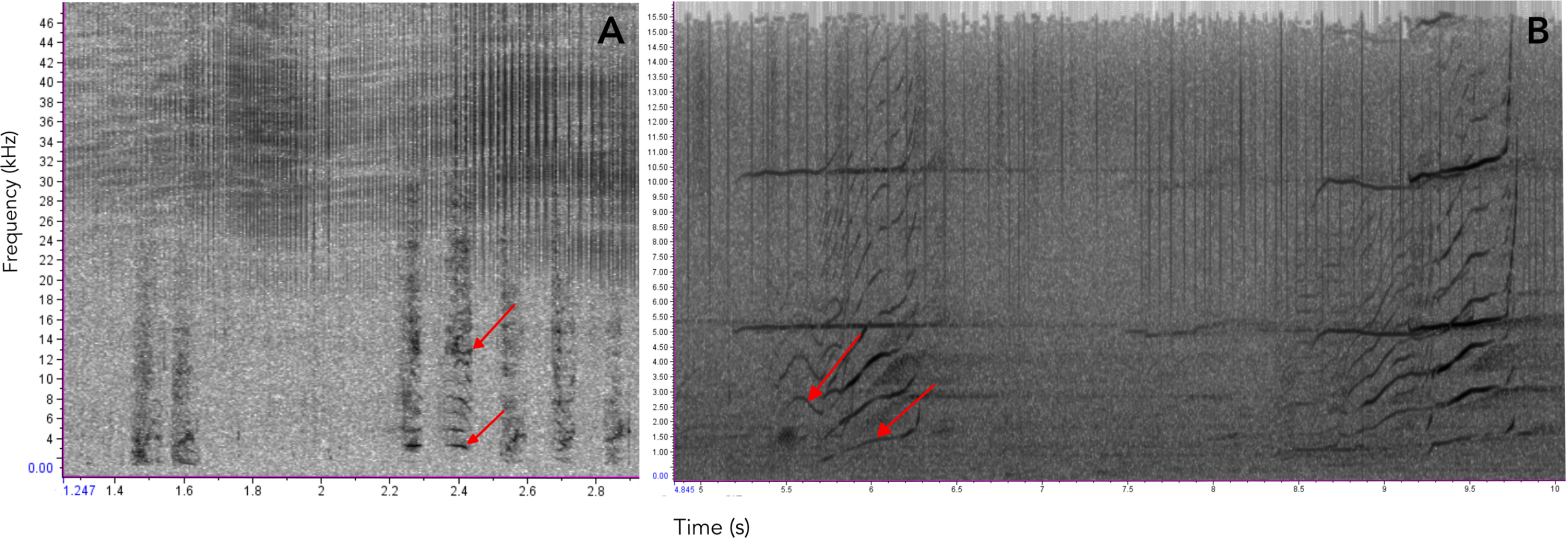
Comparison of sounds produced by Araguaian botos and orcas. Spectrograms comparing the acoustic structure of two-component pulsed calls produced by (A) Araguaian botos and (B) orcas. The two components of both calls are indicatd by red arrows. Calls from orcas were downloaded from NOOA free database on https://www.nefsc.noaa.gov/psb/acoustics/sounds.html.

Several species of toothed whales emit calls of similar acoustic nature as the ones described here for botos (Filatova et al., 2012; Fitch, Neubauer & Herzel, 2002; Ford, 1989; Deecke, Ford & Spong, 1999; Deecke et al., 2010; Deecke et al., 2011; Garland, Castellote & Berchok, 2015; Marcoux, Auger-Méthé & Humphries, 2012; Miller & Bain, 2000; Papale et al., 2015; Pérez et al., 2017; Sjare & Smith, 1986; Vergara & Barrett-Lennard, 2008; Vergara, Michaud & Barrett-Lennard, 2010; Yurk et al., 2002; Zwamborn & Whitehead, 2017. For example, the calls of orcas (*Orcinus orca*) and pilot whales (*Globicephala* spp.) have been shown to contain non-linear features suggesting they may carry information on group identity and maintaining social cohesion (Deecke et al., 2010; Pérez et al., 2017; Yurk et al., 2002; Zwamborn & Whitehead, 2017) (see Fig. 9 for an example). Similarly, Marcoux, Auger-Méthé & Humphries (2012) show evidence that narwhal (*Monodon* Monoceros) calls might be related to specific groups or individuals. Non-linear calls have also been reported to convey individuals’ identity and/or emotional state (Fitch, Neubauer & Herzel, 2002; Papale et al., 2015). Given these similarities we propose these two-component signals could have evolved early in the evolutionary history of toothed whales as social contact signals, likely for mother-calf interactions and later in the lineage leading to delphinids it evolved into a group recognition signal.

We recommend that future studies analyse recordings of non-habituated botos, to verify possible differences in the acoustic behaviour of human-habituated and non-human habituated animals. Nevertheless, the botos recorded in this study are free-ranging animals that interact with other members of their population when not in the Mocajuba market, and therefore their sounds are likely representative of their species.

## Conclusions

We show that the acoustic repertoire of botos is diverse and includes a wealth of signal types. The Araguaian river dolphins studied at Mocajuba fish market produce a diverse acoustic repertoire, as we found 237 sound-types, mostly pulsed calls, and our analysis indicate that there is more to discover. Notwithstanding, these sounds are mostly complex in structure presenting nonlinear phenomena. The animals we studied are habituated to humans, which provided a unique opportunity to shed light on the acoustic and social behaviour of this understudied species. Under relatively controlled conditions we identified more than half of the studied animals and recorded their acoustic and underwater behaviour. When possible, we matched recordings with video footage of calves as they reunited with their mothers. During these reunions calves appeared to use the two-component calls as contact calls, nevertheless further investigation is needed to understand the importance of these calls for mother-calf interactions. Given that Araguaian river dolphin pulsed calls are similar in acoustic structure to those of delphinids, we propose that these signals could have evolved early in the evolutionary history of toothed whales as social calls, likely as mother-calf contact calls, and that later in the lineage leading to dolphins its function evolved to group/family call recognition, though this needs to be tested in future studies. Furthermore, studies in areas where dolphins are not human-habituated should be conducted in order to verify possible differences in the acoustic behaviour of Araguaian botos with and without direct interactions with humans.

##  Supplemental Information

10.7717/peerj.6670/supp-1 Audio S1 Short two-component calls produced by an Araguaian boto calf

10.7717/peerj.6670/supp-2 Audio S2 Short calls with subharmonics produced by an Araguaian boto

10.7717/peerj.6670/supp-3 Audio S3 Short call with no non-linear phenomena produced by an Araguaian boto

10.7717/peerj.6670/supp-4 Audio S4 Tonal whistle produced by an Araguaian boto and the longest sound in our recordings

## Additional Information and Declarations

### Data Availability

The following information was supplied regarding data availability: Data is available at Figshare: Melo-Santos, Gabriel; Rodrigues, Angélica Lúcia Figueiredo; Tardin, Rodrigo; Maciel, Israel; Marmontel, Miriam; da Silva, Maria Luisa; et al. (2019): Market boto videos (Supplementary Material). figshare. Fileset. DOI: 10.6084/m9.figshare.6459317.v1. Melo-Santos, Gabriel; Rodrigues, Angélica Lúcia Figueiredo; Tardin, Rodrigo; Maciel, Israel; Marmontel, Miriam; da Silva, Maria Luisa; et al. (2019): Data tables for Inia araguaiaensis acoustic signals. figshare. Dataset. DOI: 10.6084/m9.figshare.7862192.v1.

## References

[ref-1] Amorim TOS, Andriolo A, Reis SS, Dos Santos ME (2016). Vocalizations of Amazon river dolphins (*Inia geoffrensis*): characterization, effect of physical environment and differences between populations. The Journal of the Acoustical Society of America.

[ref-2] Au WW (2000). Hearing in whales and dolphins: an overview. Hearing by whales and dolphins.

[ref-3] Bebus SE, Herzing DL (2015). Mother-offspring signature whistle similarity and patterns of association in Atlantic spotted dolphins (*Stenella frontalis*). Animal Behavior and Cognition.

[ref-4] Best RC, Da Silva VM, Perrin WF, Brownell Jr RL, Kaiya Z, Jionkang L (1989). Amazon river dolphin, boto *Inia geoffrensis* (de Blainville, 1817). Handbook of marine mammals. River Dolphins Larg. Toothed Whales.

[ref-5] Best RC, Da Silva VM (1993). Inia geoffrensis. Mammalian Species.

[ref-6] Boisseau O (2005). Quantifying the acoustic repertoire of a population: the vocalizations of free-ranging bottlenose dolphins in Fiordland, New Zealand. The Journal of the Acoustical Society of America.

[ref-7] Brownell RL, Herald ES (1972). Lipotes vexillifer. Mammalian Species.

[ref-8] Caldwell MC, Caldwell DK, Evans WE (1966). Sounds and behavior of captive Amazon freshwater dolphins, Inia geoffrensis.

[ref-9] Cassens I, Vicario S, Waddell VG, Balchowsky H, Van Belle D, Ding W, Fan C, Mohan RL, Simões Lopes PC, Bastida R, Meyer A, Stanhope MJ, Milinkovitch MC (2000). Independent adaptation to riverine habitats allowed survival of ancient cetacean lineages. Proceedings of the National Academy of Sciences of the United States of America.

[ref-10] Clark CW (1994). Blue deep voices: insights from the Navy’s Whales’ 93 program. Whalewatcher.

[ref-11] Clark CW (1995). Application of US Navy underwater hydrophone arrays for scientific research on whales. Reports International Whaling Commission.

[ref-12] Cremer MJ, Holz AC, Bordino P, Wells RS, Simões Lopes PC (2017). Social sounds produced by franciscana dolphins, *Pontoporia blainvillei* (Cetartiodactyla, Pontoporiidae). The Journal of the Acoustical Society of America.

[ref-13] Crespo EA, Harris G, González R (1998). Group size and distributional range of the franciscana, *Pontoporia blainvillei*. Marine Mammal Science.

[ref-14] Da Silva VMF, Perrin WF, Wursig B, Thewissen JGM (2009). Amazon river dolphin: *Inia geoffrensis*. Encyclopedia of marine mammals (Second Edition).

[ref-15] Deecke VB, Barrett-Lennard LG, Spong P, Ford JK (2010). The structure of stereotyped calls reflects kinship and social affiliation in resident killer whales (*Orcinus orca*). Naturwissenschaften.

[ref-16] Deecke VB, Ford JK, Spong P (1999). Quantifying complex patterns of bioacoustic variation: use of a neural network to compare killer whale (*Orcinus orca*) dialects. The Journal of the Acoustical Society of America.

[ref-17] Deecke VB, Janik VM (2006). Automated categorization of bioacoustic signals: avoiding perceptual pitfalls. The Journal of the Acoustical Society of America.

[ref-18] Deecke VB, Nykänen M, Foote AD, Janik VM (2011). Vocal behaviour and feeding ecology of killer whales *Orcinus orca* around Shetland, UK. Aquatic Biology.

[ref-19] Diazgranados MC, Trujillo F (2002). Vocal repertoire of the freshwater dolphins *Inia geoffrensis* and *Sotalia fluviatilis* in Colombia, South America. The Journal of the Acoustical Society of America.

[ref-20] Ding W, Würsig B, Evans W (1995). Comparisons of whistles among seven odontocete species. Sensory systems of aquatic mammals.

[ref-21] Ding W, Würsig B, Leatherwoods S (2001). Whistles of boto, *Inia geoffrensis*, and tucuxi, *Sotalia fluviatilis*. The Journal of the Acoustical Society of America.

[ref-22] Dos Santos GMA, Quaresma AC, Barata RR, Martins BM, Siciliano S, e Silva JDS, Emin-Lima R (2012). Etho-ecological study of the Amazon River dolphin, *Inia geoffrensis* (Cetacea: Iniidae), and the dolphins of the genus *Sotalia* (Cetacea: Delphinidae) in Guamá River, Amazonia. Marine Biodiversity Records.

[ref-23] Dos Santos GMA, Rodrigues ALF, Arcoverde DL, Ramos I, Sena L, Silva ML (2014). Unusual records of the behavior of boto *Inia* sp.(Cetartiodactyla, Iniidae) in the lower reaches of the Tocantins and Guamá River, Amazônia. Dolphins: ecology, behavior and conservation strategies.

[ref-24] Filatova OA, Deecke VB, Ford JK, Matkin CO, Barrett-Lennard LG, Guzeev MA, Burdin AM, Hoyt E (2012). Call diversity in the North Pacific killer whale populations: implications for dialect evolution and population history. Animal Behaviour.

[ref-25] Fitch WT, Neubauer J, Herzel H (2002). Calls out of chaos: the adaptive significance of nonlinear phenomena in mammalian vocal production. Animal Behaviour.

[ref-26] Ford JK (1989). Acoustic behaviour of resident killer whales (*Orcinus orca*) off Vancouver Island, British Columbia. Canadian Journal of Zoology.

[ref-27] Fripp D (2005). Bubblestream whistles are not representative of a bottlenose dolphin’s vocal repertoire. Marine Mammal Science.

[ref-28] Garland EC, Castellote M, Berchok CL (2015). Beluga whale (*Delphinapterus leucas*) vocalizations and call classification from the eastern Beaufort Sea population. The Journal of the Acoustical Society of America.

[ref-29] Gomez-Salazar C, Trujillo F, Portocarrero-Aya M, Whitehead H (2012). Population, density estimates, and conservation of river dolphins (*Inia* and *Sotalia*) in the Amazon and Orinoco river basins. Marine Mammal Science.

[ref-30] Gomez-Salazar C, Trujillo F, Whitehead H (2012). Ecological factors influencing group sizes of river dolphins (*Inia geoffrensis* and *Sotalia fluviatilis*). Marine Mammal Science.

[ref-31] Goulding M, Barthem R, Ferreira EJG, Duenas R (2003). The Smithsonian atlas of the Amazon.

[ref-32] Hamilton H, Caballero S, Collins AG, Brownell RL (2001). Evolution of river dolphins. Proceedings of the Royal Society of London B: Biological Sciences.

[ref-33] Hrbek T, Da Silva VMF, Dutra N, Gravena W, Martin AR, Farias IP (2014). A new species of river dolphin from Brazil or: how little do we know our biodiversity. PLOS ONE.

[ref-34] Jensen FH, Rocco A, Mansur RM, Smith BD, Janik VM, Madsen PT (2013). Clicking in shallow rivers: short-range echolocation of Irrawaddy and Ganges river dolphins in a shallow, acoustically complex habitat. PLOS ONE.

[ref-35] Jones BL (2014). The ontogeny of whistle production in infant Atlantic Bottlenose Dolphins (Tursiops truncatus) during the first thirty days of life. Masters Thesis.

[ref-36] Junk WJ, Piedade MTF, Schöngart J, Cohn-Haft M, Adeney JM, Wittmann F (2011). A classification of major naturally-occurring Amazonian lowland wetlands. Wetlands.

[ref-37] Kamminga C, Van Hove MT, Englesma FJ, Terry RP (1993). Investigations on cetacean sonar X: a comparative analysis of underwater echolocation clicks of *Inia* spp. and *Sotalia* spp. Aquatic Mammals.

[ref-38] Lammers MO, Au WW, Herzing DL (2003). The broadband social acoustic signaling behavior of spinner and spotted dolphins. The Journal of the Acoustical Society of America.

[ref-39] Leatherwoods S, Reeves RR (1994). River dolphins: a review of activities and plans of the Cetacean Specialist Group. Aquatic Mammals.

[ref-40] Magurran AE (2004). Measuring biological diversity.

[ref-41] Mann J (1999). Behavioral sampling methods for cetaceans: a review and critique. Marine Mammal Science.

[ref-42] Marcoux M, Auger-Méthé M, Humphries MM (2012). Variability and context specificity of narwhal (*Monodon monoceros*) whistles and pulsed calls. Marine Mammal Science.

[ref-43] Martin AR, Da Silva VM (2004). Number, seasonal movements, and residency characteristics of river dolphins in an Amazonian floodplain lake system. Canadian Journal of Zoology.

[ref-44] Martin AR, Da Silva VMF, Rothery P (2008). Object carrying as socio-sexual display in an aquatic mammal. Biology Letters.

[ref-45] Martin AR, Da Silva V, Salmon DL (2004). Riverine habitat preferences of botos (*Inia geoffrensis*) and tucuxis (*Sotalia fluviatilis*) in the central Amazon. Marine Mammal Science.

[ref-46] May-Collado LJ, Agnarsson I (2011). Phylogenetic analysis of conservation priorities for aquatic mammals and their terrestrial relatives, with a comparison of methods. PLOS ONE.

[ref-47] May-Collado LJ, Wartzok D (2007). The freshwater dolphin *Inia geoffrensis* geoffrensis produces high frequency whistles. The Journal of the Acoustical Society of America.

[ref-48] May-Collado LJ, Wartzok D (2009). A characterization of Guyana dolphin (*Sotalia guianensis*) whistles from Costa Rica: the importance of broadband recording systems. The Journal of the Acoustical Society of America.

[ref-49] Miller PJ, Bain DE (2000). Within-pod variation in the sound production of a pod of killer whales, *Orcinus orca*. Animal Behaviour.

[ref-50] Mizue KA, Nishiwaki MA, Takemura AK (1971). The underwater sound of Ganges river dolphins (*Platanista gangetica*). The Scientific Reports of the Whales Research institute.

[ref-51] Papale E, Buffa G, Filiciotto F, Maccarrone V, Mazzola S, Ceraulo M, Giacoma C, Buscaino G (2015). Biphonic calls as signature whistles in a free-ranging bottlenose dolphin. Bioacoustics.

[ref-52] Pavanato HJ, Melo-Santos G, Lima DS, Portocarrero-Aya M, Paschoalini M, Mosquera F, Trujillo F, Meneses R, Marmontel M, Maretti C (2016). Risks of dam construction for South American river dolphins: a case study of the Tapajós River. Endangered Species Research.

[ref-53] Penner RH, Murchison AE (1970). Experimentally demonstrated echolocation in the Amazon River porpoise, Inia geoffrensis (Blainville).

[ref-54] Pérez JM, Jensen FH, Rojano-Doñate L, Aguilar de Soto N (2017). Different modes of acoustic communication in deep-diving short-finned pilot whales (*Globicephala macrorhynchus*). Marine Mammal Science.

[ref-55] Pilleri G, Gihr M (1977). Observations on the Bolivian, *Inia boliviensis*, (D’Orbigny, 1834) and the Amazonian bufeo, *Inia geoffrensis* (Blainville, 1817), with a description of a new subspecies (*Inia geoffrensis humboldtiana*). Investigations on CETACEA.

[ref-56] Podos J, Da Silva VM, Rossi-Santos MR (2002). Vocalizations of Amazon river dolphins, *Inia geoffrensis*: insights into the Evolutionary origins of delphinid whistles. Ethology.

[ref-57] R Core Team (2018). https://www.R-project.org/.

[ref-58] Reeves RR, Martin AR, Perrin WF, Wursig B, Thewissen JGM (2009). River dolphins. Encyclopedia of marine mammals (Second Edition).

[ref-59] Ribeiro MCLDB, Petrere M, Juras AA (1995). Ecological integrity and fisheries ecology of the Araguaia—Tocantins River Basin, Brazil. River Research and Applications.

[ref-60] Rodrigues ALF, Melo-Santos G, Ramos-Santos I, Andrade AM, Arcoverde DL, Sena L, Da Silva ML (2019). Interactions between children, teenagers and botos (Inia araguaiaensis and Inia geoffrensis) in markets and fairs of Eastern Amazon. Ocean & Coastal Management.

[ref-61] Sjare BL, Smith TG (1986). The vocal repertoire of white whales, *Delphinapterus leucas*, summering in Cunningham Inlet, Northwest Territories. Canadian Journal of Zoology.

[ref-62] Smolker RA, Mann J, Smuts BB (1993). Use of signature whistles during separations and reunions by wild bottlenose dolphin mothers and infants. Behavioral Ecology and Sociobiology.

[ref-63] Sugimatsu H, Kojima J, Ura T, Bahl R, Sagar VS, Chauhan R (2017). Real-time automatic estimation of the number of migrating Ganges river dolphins (*Platanista gangetica*) during the acoustic census by using a mobile four-hydrophone array system.

[ref-64] Tokuda I, Riede T, Neubauer J, Owren MJ, Herzel H (2002). Nonlinear analysis of irregular animal vocalizations. The Journal of the Acoustical Society of America.

[ref-65] Tyack PL, Mann J, Connor RC, Tyack PL, Whitehead H (2000). Functional aspects of cetacean communication. Cetacean societies: field studies of dolphins and whales.

[ref-66] Vergara V, Barrett-Lennard LG (2008). Vocal development in a beluga calf (*Delphinapterus leucas*). Aquatic Mammals.

[ref-67] Vergara V, Michaud R, Barrett-Lennard L (2010). What can captive whales tell us about their wild counterparts? Identification, usage, and ontogeny of contact calls in belugas (*Delphinapterus leucas*). International Journal of Comparative Psychology.

[ref-68] Wang D, Wang KX, Akamatsu TK (1999). Study on whistling of the Chinese river dolphin (*Lipotes vexillifer*). Oceanologia et Limnologia Sinica.

[ref-69] Wang K, Wang D, Akamatsu T, Fujita K, Shiraki R (2006). Estimated detection distance of a baiji’s (Chinese river dolphin, *Lipotes vexillifer*) whistles using a passive acoustic survey method. The Journal of the Acoustical Society of America.

[ref-70] Wang D, Wenxiang L, Zhifan W (1995). A preliminary study of the acoustic behavior of the baiji, *Lipotes vexillifer*. Biology and conservation of the river dolphins.

[ref-71] Wilden I, Herzel H, Peters G, Tembrock G (1998). Subharmonics, biphonation, and deterministic chaos in mammal vocalization. Bioacoustics.

[ref-72] Xiao Y, Jing R (1989). Underwater acoustic signals of the baiji, *Lipotes vexillifer*. Biology and Conservation of the River Dolphins, Occasional paper.

[ref-73] Yurk H, Barrett-Lennard L, Ford JKB, Matkin CO (2002). Cultural transmission within maternal lineages: vocal clans in resident killer whales in southern Alaska. Animal Behaviour.

[ref-74] Zhou K, Perrin WF, Wursig B, Thewissen JGM (2009). Baiji (Lipotes vexillifer). Encyclopedia of marine mammals.

[ref-75] Zwamborn EM, Whitehead H (2017). Repeated call sequences and behavioural context in long-finned pilot whales off Cape Breton, Nova Scotia, Canada. Bioacoustics.

